# A Rare Case of Relapsed Sarcoidosis Presenting As Severe Thrombocytopenia Associated With Intracerebral Hemorrhage Due to Bone Marrow Involvement

**DOI:** 10.7759/cureus.37973

**Published:** 2023-04-22

**Authors:** Prabasha Weeraddana, Hedaya Othman, Teena Thomas, Malsha Walgamage, Oluwole Odujoko, Wenli Gao

**Affiliations:** 1 Internal Medicine, Danbury Hospital, Danbury, USA; 2 Pathology and Laboratory Medicine, Danbury Hospital, Danbury, USA; 3 Oncology, Danbury Hospital, Danbury, USA

**Keywords:** bone marrow sarcoidosis, granulomatous disease, petechiae rash, relapsed sarcoidosis, bone marrow involvement, intracerebral hemorrhage, severe thrombocytopenia, sarcoidosis

## Abstract

Sarcoidosis is a systemic granulomatous disease characterized by the hyperactivation of CD4 T cells, CD8 T cells, and macrophages. Clinical presentations of sarcoidosis are highly variable. Sarcoidosis is unknown in its etiology, but it suggests it may result from exposure to specific environmental agents in genetically susceptible people. Sarcoidosis commonly involves the lungs and lymphoid system. Bone marrow involvement in sarcoidosis is uncommon. Sarcoidosis rarely results in intracerebral hemorrhage due to severe thrombocytopenia secondary to bone marrow involvement. We present the case of a 72-year-old woman who has been in remission from sarcoidosis for 15 years and developed intracerebral hemorrhage secondary to severe thrombocytopenia due to sarcoidosis recurrence in the bone marrow. The patient presented to the emergency department with a generalized, non-blanching petechiae rash and nose and gum bleeding. Her labs showed a platelet count of less than 10.000/mcL, and computed tomography (CT) showed intracerebral hemorrhage. A bone marrow biopsy revealed a small, non-caseating granuloma indicative of a sarcoidosis relapse in the bone marrow.

## Introduction

Sarcoidosis is a systemic inflammatory disorder that triggers granuloma formation in multiple organs with an unclear etiology [1]. The prevalence of sarcoidosis varies depending on the country of the world, from 1-5 per 100,000 in South Korea to 140-160 per 100,000 in Sweden [2]. Only 10% of sarcoidosis involves extrapulmonary manifestations that can cause significant mortality and morbidity. Bone marrow sarcoidosis (BMS) accounts for 1%-13% of cases and is much less reported [2,3]. Hematological manifestations such as thrombocytopenia are rare extrapulmonary complications (<1%) and have been rarely reported in sarcoidosis cases. This condition could be severe but usually follows a favorable course without death or severe bleeding [4]. Intracranial hemorrhage (ICH) is a rare and serious adverse effect of severe thrombocytopenia that can cause death or significant morbidity [5]. This case presented a 72-year-old woman with a relapse of sarcoidosis in the bone marrow who initially presented with severe thrombocytopenia complicated by intracerebral hemorrhage.

## Case presentation

A 72-year-old woman with a past medical history of chronic obstructive pulmonary disease (COPD), a history of breast cancer with status post-radiation therapy, pulmonary sarcoidosis, and liver cirrhosis presented to the emergency department (ED) with intermittent nose bleeding, gum bleeding, and bright red blood per rectum for one week. She also complained of a progressive rash that started in her legs and spread to her torso, arms, and face for two days. She denied fever, weight loss, night sweats, and a loss of appetite. She had cancer on the right breast and completed five years of tamoxifen therapy following a lumpectomy and adjuvant radiotherapy. She was diagnosed with pulmonary sarcoidosis via bronchoscopy biopsy 25 years ago. She remained disease-free and had not been on any treatment for the last 15 years. She was treated with steroids for a brief period initially, but never with steroid-sparing drugs. She was regularly followed up with a pulmonologist and underwent computerized chest tomography (CT) yearly.

On presentation to the ED, she was hypertensive with systolic blood pressure (SBP) at 150/75 mmHg and slightly tachycardic with a heart rate of 100-120 bpm. The physical examination revealed a non-blanching petechiae-like rash on the face, chest, torso, and bilateral lower extremities. The neurological examination was unremarkable. The bilateral lungs were clear, the abdomen was soft, and there was no hepatosplenomegaly.

The lab tests revealed anemia with hemoglobin of 11.3 mg/dL and thrombocytopenia with platelets less than 10.000/mcL. The liver function tests were within the normal range. The lactate dehydrogenase level was 238 U/L, and the D-dimer level was 560 ng/mL. Coagulation panels showed an activated partial thromboplastin time (APTT) of 26 seconds, a fibrinogen level of 420 mg/dL, a prothrombin time (PT) of 13.5 seconds, and an international normalized ratio (INR) of 1.06. A peripheral blood smear revealed normochromic normocytic anemia with severe thrombocytopenia, adequate leukocytes, mild neutrophilia, several bands, and reactive monocytes and lymphocytes. There were no blasts or schistocytes. There was no clumping of platelets, but there were few megakaryocytes. Kidney function, electrolytes, acid-base balance, coagulation tests, and inflammatory markers were all within normal limits. Laboratory investigations on admission are shown in Table 1

**Table 1:** Lab investigations were done on admission. WBC: white blood cells; Hg: mercury; ALT: alanine aminotransferase; AST: aspartate aminotransferase; LDH: lactate dehydrogenase; INR: international normalized ratio; aPTT: activated partial thromboplastin time; PT: prothrombin time; BUN: blood urea nitrogen; ESR: erythrocyte sedimentation rate; PTH: parathyroid hormone

Test name	Value	Normal range
WBC (103/µL)	9.9	4000-11,000
Hg (g/dL)	11.3	12.1-15.1
Platelet (103/mL)	< 10,000	150,000-450,000
AST (U/L)	28	8-33
ALT (U/L)	18	7-56
Total bilirubin (mg/dL)	0.5	0.1-1.2
INR	1.06	0.8-1.1
aPTT (s)	26	30-40
PT (s)	13.5	11-13.5
Fibrinogen (mg/dL)	420	200-400
LDH (U/L)	238	140-280
D-dimer (ng/mL)	560	0-500
Serum creatinine (mg/dL)	0.55	0.59-1.1
BUN (mg/dL)	21	6-24
Sodium level (mEq/L)	138	135-145
Potassium level (mmol/L)	4.1	3.6-5.2
Lactic acid level (mmol/L)	0.9	0.5-1
Calcium level (mg/dL)	10	8.6- 10.3
ESR (mm/hr)	2	0-29
PTH (pg/mL)	21	15-65
Albumin Level (g/dL)	4.5	3.4-5.4
Bicarbonate level (mmol/L)	34	22-32

**Figure 1:**
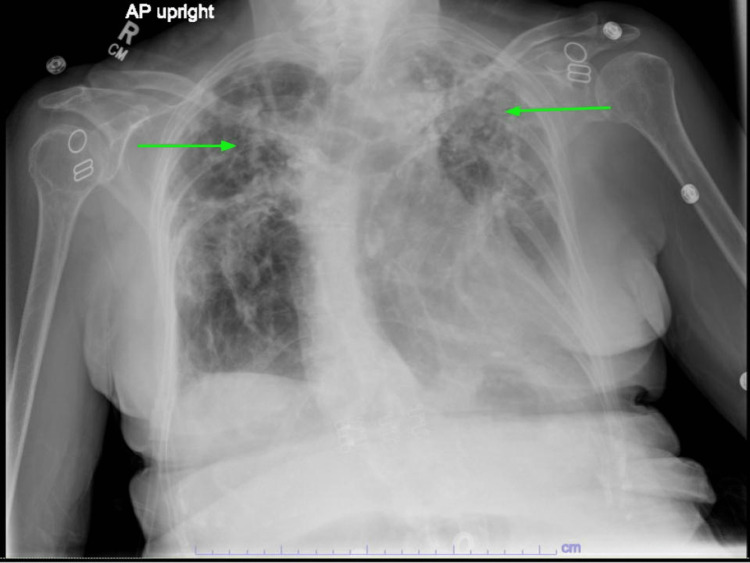
A chest x-ray showed bilateral upper lung predominant fibrotic changes (green arrows).

**Figure 2:**
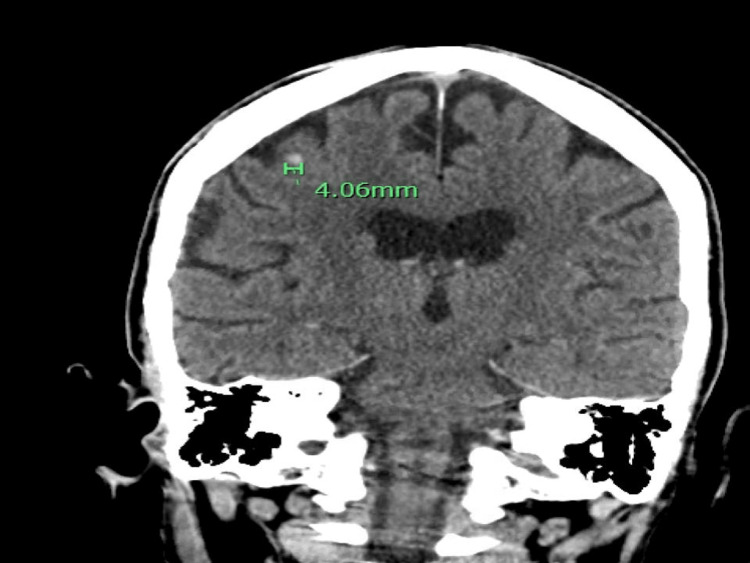
A computerized tomography (CT) of the head showed a 4 mm intracranial hemorrhage in the right frontal lobe.

Her chest x-ray showed stable bilateral upper lung predominant fibrotic changes (Figure 1). Computerized tomography (CT) of the head showed a 4 mm intracranial hemorrhage in the right frontal lobe (Figure 2). The oncologist recommended platelet transfusion with a platelet level goal of 10,000/mcL and an interventional radiologically-guided bone marrow (BM) biopsy. Neurosurgery did not recommend acute neurosurgical intervention, and she was admitted to the intensive care unit (ICU) for neurological monitoring.

**Figure 3:**
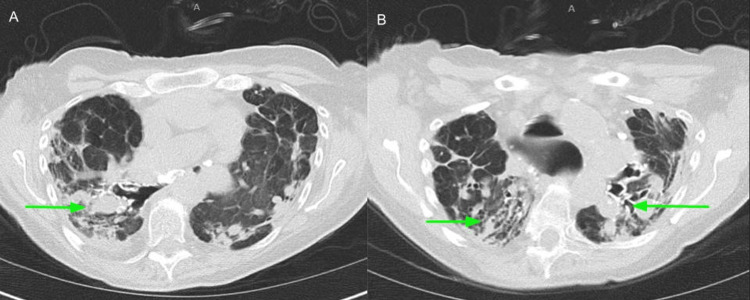
A chest CT without contrast showed extensive scarring and fibrosis within the apical prominence (green arrows pointed to scarring and fibrosis; A, B: axial views at different sections of the lungs).

**Figure 4:**
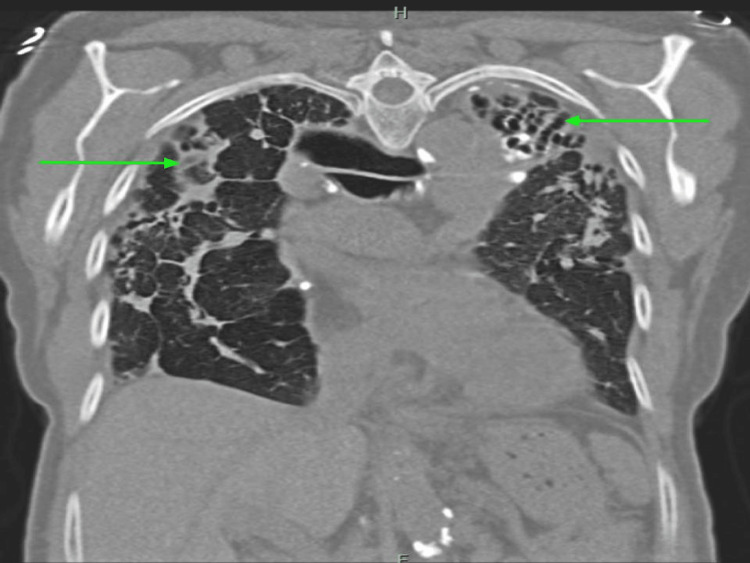
CT chest without contrast (coronal view) showed extensive scarring and fibrosis within apical prominence (green arrows pointing to scarring/fibrosis).

She underwent further investigation for thrombocytopenia. Her serum protein electrophoresis (SPEP) showed an isolated elevation of alpha-2 globulins suggestive of acute inflammation. She had low levels of vitamin D 25 OH at 23 ng/ml (30-100 ng/ml). Ultrasonography of the abdomen showed no hepatosplenomegaly. A CT chest without contrast revealed stable bronchiectasis, most prominent at the left upper lobe, with no appreciable change in the extensive scarring and fibrosis within the apical prominence compared to last year's imaging (Figures 3-4).

**Figure 5:**
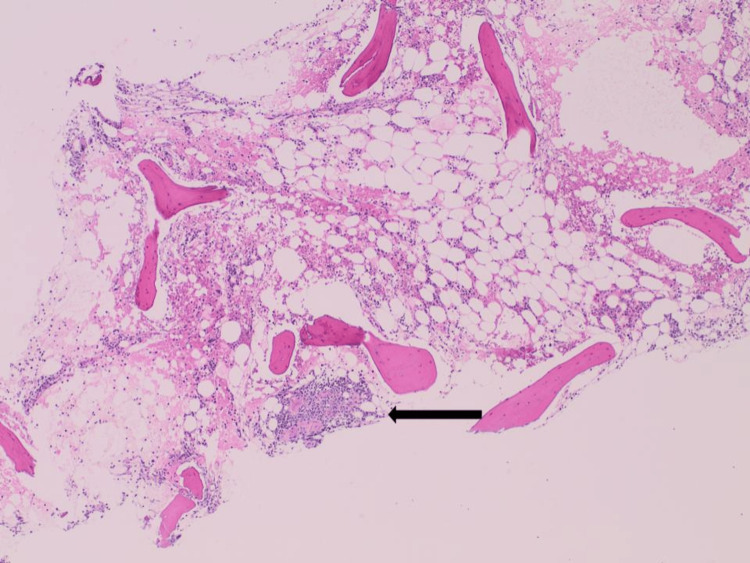
A section of the bone marrow biopsy (H and E x4) showed a small focus of non-necrotizing granuloma (black arrow). H&E: hematoxylin and eosin stain

**Figure 6:**
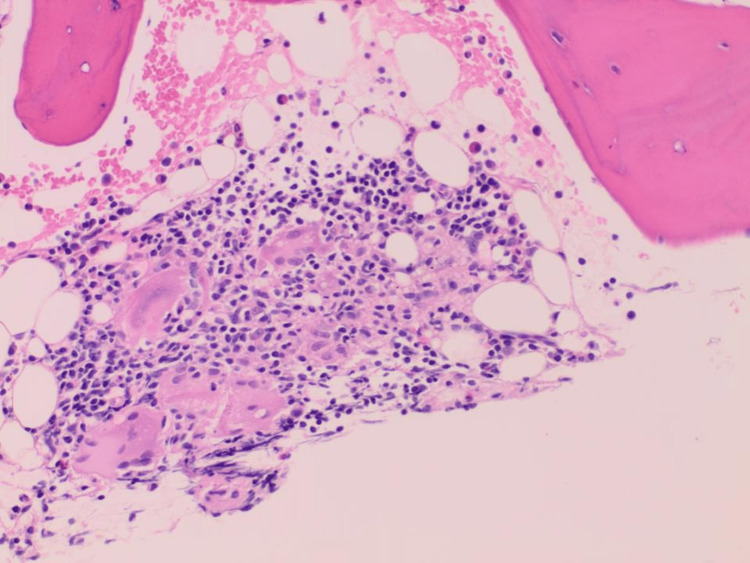
A section of the bone marrow biopsy showed the granuloma at higher magnification with epithelioid histiocytes that have fused to form giant cells surrounded by mononuclear lymphocytes (H and E x20).

A bone marrow biopsy revealed normocellular marrow showing maturing trilineage hematopoiesis and a small non-necrotizing granuloma consistent with known sarcoidosis (Figures 5-6).

It was negative for diagnostic evidence of dysplasia, lymphoproliferative disorder, plasma cell neoplasm, or acute leukemia. Ziehl-Neelsen staining for acid-fast bacilli and Grocott methenamine silver (GMS) staining for fungal organisms were negative. Her blood and urine cultures were also negative. Flow cytometric immunophenotypic analysis found no evidence of abnormal myeloid maturation or an increased blast population; there was no evidence of a lymphoproliferative disorder. She also had negative results for antinuclear antibody, Lyme panel, human immunodeficiency virus (HIV) antigen and antibodies, hepatitis C and B, Anaplasia, and Babesia.

After the BM biopsy, she was started on prednisone 50 mg daily and was administered a dose of intravenous immunoglobulins at 1 mg/kg (50 mg). She also received multiple platelet transfusions. The patient was discharged with prednisone, tapering off once platelets stabilized

## Discussion

Sarcoidosis is a granulomatous disease that usually affects the lungs and extrapulmonary organs, including the eyes, skin, liver, spleen, and lymph nodes [6]. Sarcoidosis is still a disease with an unknown etiology. Mechanisms underlying granuloma formation include genetic susceptibility and environmental factors [7]. The clinical manifestations of sarcoidosis are various and depend on the organ involved. Common symptoms include a persistent dry cough, eye and skin manifestations, weight loss, fatigue, night sweats, and erythema nodosum [6]. Our patient presented with bleeding from the gums, nose bleeding, intracerebral hemorrhage (ICH), and a petechial rash, which are uncommon presentations of sarcoidosis. On admission, she had severe thrombocytopenia with a platelet count of less than 10,000/ml. Despite having had sarcoidosis in the past, a relapse was not initially suspected as the patient had been in remission for 15 years.

Thrombocytopenia can be due to decreased platelet production, increased platelet sequestration, destruction, or consumption [8]. In our case, the patient developed a new onset of thrombocytopenia. As thrombocytopenia is associated with a variety of conditions, diagnosis is challenging. The new onset of thrombocytopenia requires urgent investigation, especially in life-threatening situations such as our case of ICH. To our knowledge, no other cases of ICH have been reported following severe thrombocytopenia resulting from sarcoidosis involvement of the bone marrow.

There are many causes of thrombocytopenia. It can be infectious, such as hepatitis, HIV, parvovirus B19, malaria, sepsis, or inflammatory or autoimmune, such as disseminated intravascular coagulation, hemolytic uraemic syndrome, bone marrow failure from acute leukemias, or a congenital defect [9]. Here, we performed a coagulation panel and excluded disseminated intravascular coagulation (DIC), one of the most life-threatening causes of thrombocytopenia. There was low suspicion of thrombotic thrombocytopenic purpura (TTP), with a plasmic score of three points. We excluded splenomegaly with platelet sequestration by ultrasonography (US) of the abdomen. In addition to negative anaplasmosis, Babesia, human immunodeficiency virus (HIV), and hepatitis panels, she had negative blood and urine cultures and a negative bone marrow biopsy for acute leukemia or lymphoproliferative disorders.

Severe thrombocytopenia leading to ICH is rare. This patient had ICH secondary to severe thrombocytopenia, and luckily, she had no neurological symptoms. The incidence of ICH in patients with thrombocytopenia due to immune thrombocytopenia (ITP) was 1.540% in all ages [10], and with leukemia, it was 3.5% [11].

This patient suffers from relapsed sarcoidosis with bone marrow involvement. Bone marrow involvement in relapsed sarcoidosis is rare, and Jungmin Lee reported one case [12].

Sarcoidosis is a diagnostic challenge. There is no universally accepted standardized method for diagnosis, but it is based on three major criteria. The criteria are based on clinical and/or radiological presentation, histological evidence of non-necrotizing granulomatous inflammation in one or more tissues, and the exclusion of alternative causes of granulomatous disease [13]. Non-necrotizing granulomas are not specific to sarcoidosis. Several conditions cause non-necrotizing granulomas in the bone marrow. Medications such as adalimumab induce non-caseating granulomas in the bone marrow in patients with rheumatoid arthritis [14]. Autoimmune disorders such as rheumatoid arthritis (RA) and malignancies such as Hodgkin’s lymphoma, infections such as Coxiella burnetii, Ebstein-Barr virus infection, leishmaniasis, histoplasmosis, Bartonellosis, mycobacterial diseases, and Brucellosis can lead to the formation of granulomas in the BM [15]. Therefore, the diagnosis of sarcoidosis requires a complete history and physical examination. Where indicated, additional tests aim to exclude other disorders, particularly those that produce granulomas. We performed a bone marrow biopsy on this patient. It was negative for lymphoproliferative disorder, plasma cell neoplasm, acute leukemia, acid-fast bacilli, and Grocott methenamine silver (GMS) staining for fungi.

The first-line treatment for sarcoidosis is corticosteroids, but they have several serious adverse effects. Therefore, corticosteroids are indicated only when symptoms appear or cause life- or organ-threatening disease. Second-line therapy for sarcoidosis consists of disease-modifying antirheumatic drugs (DMARDs). Second-line therapies are indicated when patients’ conditions worsen using corticosteroids, such as decompensated heart failure, uncontrolled diabetes, severe obesity, uncontrolled hypertension, and glaucoma. Third-line therapy for sarcoidosis involves biological agents. These are recommended for patients with refractory disease who do not respond to glucocorticoids and DMARDs or cannot tolerate these agents [16-17]. Our patient was successfully treated with steroids, even though extrapulmonary sarcoidosis usually requires immunosuppression.

## Conclusions

Bone marrow involvement in sarcoidosis is rare. Clinical presentations of sarcoidosis are diverse, and bleeding manifestations are an uncommon presentation of sarcoidosis. Intracranial hemorrhage secondary to severe thrombocytopenia can be life-threatening and requires urgent evaluation for the underlying cause. Here we present a case of relapsed sarcoidosis in the bone marrow leading to intracranial hemorrhage (ICH) due to severe thrombocytopenia. Clinicians need to be aware of these rare manifestations and maintain high susceptibility to reach the diagnosis earlier, especially when encountering a patient with a history of past sarcoidosis.
